# Burden of hemodialysis on health-related quality of life: insights from a multi-center cross-sectional analysis in Southern Albania

**DOI:** 10.3389/fmed.2025.1557063

**Published:** 2025-06-10

**Authors:** Rezarta Lalo, Fatjona Kamberi, Eda Stasa, Kenedia Lalo

**Affiliations:** ^1^Department of Health Care, Faculty of Health, University of Vlora "Ismail Qemali", Vlorë, Albania; ^2^Scientific Research Centre for Public Health, University of Vlore “Ismail Qemali”, Vlorë, Albania; ^3^Faculty of Nature and Humanities Sciences, University of Korca “Fan Noli”, Korçë, Albania; ^4^Faculty of Pharmacy, College University Reald, Vlorë, Albania

**Keywords:** chronic kidney disease, haemodialysis, quality of life, clinical and socio-demographic factors, Southern Albania

## Abstract

**Background:**

Chronic renal failure represents an escalating public health issue globally, including in Albania, due to its substantial impact on morbidity and mortality rates. Since it requires ongoing treatments, such as hemodialysis, the quality of life (QOL) of affected individuals is often severely compromised. Consequently, this study was conducted to assess the burden of hemodialysis on health-related quality of life (HRQOL) and identify the associated factors.

**Methods:**

This is a multicenter, cross-sectional study conducted across three dialysis units through a public-private hemodialysis partnership, covering three major regions of Southern Albania. The Kidney Disease Quality of Life Questionnaire – Short Form (KDQOL-SF) was used for data collection. The data were processed using the statistical software IBM SPSS Statistics for Windows, Version 23.0. Binary logistic regression was used to assess patients’ quality of life in relation to various sociodemographic and clinical factors. A *p*-value ≤0.05 was considered statistically significant.

**Results:**

The mean age of participants was 56.1 ± 12.37 years. 97% of patients undergoing hemodialysis had hypertension as a comorbidity and 99% were anemic. The mean scores for the physical and mental component summaries were 34.17 ± 12.99 and 47.52 ± 13.95, respectively. Regarding the overall quality of life score, our findings revealed that females (39.6 ± 8.7), older individuals (42.2 ± 10.4), married patients (44.8 ± 9.9), and those with three or more comorbidities (42.5 ± 9.0) had the lowest scores, indicating the worst quality of life.

**Conclusion:**

The data suggest that, age, gender, civil status and number of co-morbidities are significant factors influencing the overall quality of life of hemodialysis patients. Participants with multiple comorbidities, particularly older, female, married, reported the lowest quality of life scores. These findings indicate that such factors may contribute to poorer health outcomes, suggesting the need for tailored interventions by healthcare professionals to reduce the burden of hemodialysis on health-related quality of life.

## Introduction

Kidney failure, the most advanced stage of chronic kidney disease (CKD), has recently been identified as a leading cause of morbidity and mortality globally (1). Around 850 million people are affected by CKD, making it a significant health concern. It is predicted that by 2040, CKD will be the fifth most common chronic condition worldwide (2, 3). In Europe, the prevalence of CKD is also rising, with one in ten Europeans affected. This increase is attributed to factors such as an aging population and the growing rates of diabetes, hypertension, and obesity. Addressing CKD is complex, as it is influenced by local health issues, cultural norms, and socioeconomic conditions (4). The COVID-19 pandemic in 2020 further complicated the situation in Europe by increasing CKD-related complications and the risk of infections for vulnerable dialysis patients (5). CKD can progress to end-stage kidney disease (ESKD), at which point kidney replacement therapy, such as dialysis or transplantation, becomes necessary (6). Among the treatments, hemodialysis (HD) is commonly used but can have a significant impact on a patient’s health-related quality of life (HRQOL) (7). Research consistently shows that patients undergoing hemodialysis experience a poor quality of life (QOL) at various stages of treatment (8, 9). Several factors contribute to this decline, including the long hours spent on dialysis, challenges in accessing care, complications related to vascular access, and the overall burden of living with the disease. Furthermore, factors such as a patient’s general health, disease progression, satisfaction with healthcare services, and personal circumstances also play a role in determining QOL. In addition to physical health, social interactions, mental well-being, physical abilities, and the capacity to perform daily tasks are often significantly affected. The impact on QOL does not only influence a patient’s day-to-day feelings but also has broader implications. It can affect survival rates, the frequency of hospital admissions, and the overall progression of the disease (10, 11). Given these wide-ranging effects, measuring QOL is crucial. It not only impacts the effectiveness of treatments and health interventions but can also serve as a reliable predictor of prognosis for patients with ESKD (12, 13).

Low-and middle-income countries (LMICs) face significant challenges in providing care for patients at risk of or requiring treatment for end-stage kidney disease, leading to these populations being disproportionately affected (4). In Albania, a developing country in the South-East Europe (SEE) region, chronic kidney disease is becoming an increasingly important public health issue, yet it remains under-researched (14).

Currently, Albania is experiencing a rise in the incidence and prevalence of chronic renal diseases, with a clear trend toward progression to the terminal stage, requiring renal replacement therapy (RRT). The prevalence of RRT in Albania, in 2016, was 348 cases per million people (15). Unfortunately, referring to data from the 2023 Global Kidney Health Atlas (ISN-GKHA), the prevalence of treated kidney failure (KF) in Albania has increased and is reported at 602 per million population (pmp), with an annual incidence rate of 126 pmp (16). Historically, dialysis was provided solely in public hospitals, but with the growing number of patients, it has become increasingly difficult to meet the demand. To address this, recent health policies have made dialysis services free for patients. Additionally, through public-private partnerships, private clinics now offer dialysis services, making them more accessible and located closer to where patients live (17). Recent research indicates that there is limited scientific information on the quality of life (QOL) of patients undergoing dialysis in Albania and the factors associated with it (14, 18, 19).

Given this gap, the present study was conducted to assess the Health-Related Quality of Life (HRQOL) in hemodialysis patients from Southern Albania. Additionally, the study aims to investigate the demographic and clinical factors that may influence the QOL of this population, providing insights that could help optimize HRQOL in these patients.

## Methods

### Study area, study design and study period

The study was conducted in three major regions of Southern Albania. Patients were recruited from three dialysis units: Regional Hospital of Gjirokastra and two dialysis units with public-private partnerships in the Korca and Vlora districts. This was a multicenter cross-sectional study carried out from September to November 2023.

### Study population and sampling procedure

The study population consisted of patients undergoing hemodialysis (HD) at three dialysis units located in the districts of Vlora (*n* = 50), Korca (*n* = 45), and Gjirokastra (*n* = 50). The patients were required to meet the inclusion criteria, which included being over 18 years of age, having End-Stage Kidney Disease (ESKD) confirmed by medical records, undergoing HD therapy for more than 6 months, and attending dialysis three times a week.

A non-probability convenience sampling method was used to recruit participants. This approach aimed to recruit the maximum number of patients who met the inclusion criteria and agreed to participate. Patients from the three dialysis units were invited to join the study. Initially, 145 patients were invited to participate in the study. However, 8 patients did not meet the inclusion criteria, 11 patients declined to participate, and 14 patients did not fully complete the questionnaire. As a result, the final sample size consisted of 117 participants, yielding a response rate of 80.6%.

### Data collection

The data collection process involved face to face interviews conducted by health professionals with Master of Science degree who were not affiliated with the HD centers where data were collected. To ensure consistency and accuracy, both data collectors and supervisors underwent a training program covering study procedures, questionnaire administration, data collection methods, and ethical considerations. Additionally, clinical information was obtained from the medical records of individual patients.

The instrument for data collection contains two sections, one section with sociodemographic (age, gender, education, employment status, marital status, economic level, place of residence) and clinical (duration of dialysis, chronic conditions as comorbidity, number of comorbidities, HD access) data and the other section is the validated Albanian version of the HRQOL. The Kidney Disease Quality of Life (KDQOL-SF™) tool was used to measure patients’ quality of life (20, 21), a standard questionnaire designed for individuals with kidney disease. Three independent translators translated the questionnaire into Albanian through a forward and backward translation process. Additionally, three healthcare professionals specializing in hemodialysis (HD) patient care reviewed the translated version for accuracy, making necessary modifications based on their feedback. A pilot study involving ten patients assessed the questionnaire’s clarity and usefulness before being administered to the final study population. Based on the pilot study’s feedback, a final version of the questionnaire was developed with minor adjustments. The questionnaire contains 36 items related to general health assessment (SF-36 Health Survey), focusing specifically on a Physical Component Summary (PCS) and Mental Component Summary (MCS), as well as 43 specific items related to the Kidney Disease Component Summary (KDCS).

The SF-36 includes 8 domains and 36 items: physical functioning (10 items), role limitations due to physical problems (4 items), role limitations due to emotional problems (3 items), pain (2 items), general health perceptions (5 items), social functioning (2 items), emotional well-being (5 items), energy/fatigue (4 items), and 1 item related to health status compared with 1 year ago.

The KDCS includes 11 domains and 43 items: symptom/problem list (12 items), effects of kidney disease (8 items), burden of kidney disease (4 items), cognitive function (3 items), quality of social interaction (3 items), sexual function (2 items), sleep (4 items), social support (2 items), work status (2 items), overall health rating (1 item, scored separately), patient satisfaction (1 item), and dialysis staff encouragement (2 items). The overall health rating includes 1 item that is scored separately. The scoring procedure for the KDQOL-SF™ transforms the raw numeric values of the items into a 0–100 scale, where higher scores indicate better quality of life (22). Internal consistency reliability was assessed using Cronbach’s *α*: (1) For the kidney disease-targeted scales of KDQOL-SF™, the internal consistency exceeded 0.80. (2) For the eight scales of the SF-36, reliability estimates ranged from 0.78 to 0.92. (3) The overall Cronbach’s *α* for the questionnaire in this study was 0.89, indicating good reliability. This analysis suggested that the KDQOL-SF™ tool is both reliable and valid for assessing the quality of life in patients with kidney disease.

### Statistical analysis

The data were analyzed using the statistical software IBM SPSS Statistics for Windows, Version 23.0 (Armonk, NY: IBM Corp.). The mean and standard deviation were used to describe quantitative variables such as age, duration of dialysis, number of comorbidities, and scores for the assessment of Health-Related Quality of Life and its components. Frequency (percentage) was used for qualitative variables. The Kolmogorov–Smirnov statistical test was used to assess the normality of the data distribution, where *p*-value greater than 0.05 indicated a normal distribution. Based on this test, the variables MCS, KDCS, and Overall demonstrated a normal distribution, whereas PCS did not. For MCS, KDCS, and Overall, parametric tests such as Analysis of Variance (ANOVA) and the independent samples t-test were used to evaluate significant differences between mean scores, with a *p*-value ≤ 0.05 considered statistically significant. For PCS, non-parametric tests including the Mann–Whitney U test and the Kruskal-Wallis test were applied to evaluate significant differences between median scores, with a *p*-value ≤ 0.05 considered statistically significant.

Binary logistic regression was performed to evaluate the association between patients’ quality of life and various sociodemographic and clinical factors. A *p*-value ≤ 0.05 was considered statistically significant. The dependent variable was Quality of Life and its components. The independent variables were the sociodemographic and clinical indicators of the patients such as age, place of residence, gender, education, employment status, marital status, economic level, the presence of comorbid conditions, HD access and duration of dialysis.

For analytical purposes the Quality of Life (QOL) variable and its components, originally continuous variables ranging from 0 to 100, were categorized into two levels: poor (<50%) and good (≥50%) quality of life. This categorization was based on the mid-point of the scale, facilitating a straightforward interpretation of lower versus higher quality of life perceptions, where scores below 50% are generally indicative of reduced quality of life among patients with chronic conditions. Categorical variables with more than two levels were also recoded into binary variables to facilitate the regression analysis. For example, education was recoded as non-university (elementary, professional, or high school) versus university level, and economic status was recoded as low versus middle/high.

## Results

The sample consisted of 117 participants with a mean age of 56.42 ± 12.93 years. The majority were male (64%), married (76%), unemployed (83.8%) and 91.5% of them had a lower level of formal education (elementary, professional, or high school education). The average duration of hemodialysis was 4.67 ± 2.59 years. Most participants had anemia (99%), and 35% had three or more comorbidities, 60.7% had Arteriovenous Fistula/ Graft (AVF/AVG) (Table 1).

**Table 1:** Socio-demographic and clinical characteristics of participants.

Variables	Total number, *n* (%)
Gender	
Female	42 (35.9)
Male	75 (64.1)
Age	
≤ 60 years	71 (60.7)
>60 years	46 (39.3)
Occupation	
Unemployed	98 (83.8)
Employed	19 (16.2)
Residence	
Urban	73 (62.4)
Rural	44 (37.6)
Education status	
Elementary	45 (38.5)
High school	49 (41.9)
Professional	13 (11.1)
University	10 (8.5)
Civil status	
Not married	28 (23.9)
Married	89 (76.1)
Economic status	
Low	36 (30.8)
Middle	77 (65.7)
High	4 (3.5)
Duration of dialysis	
<5 years	67 (57.3)
>5 years	50 (42.7)
Comorbidity	
Diabetic	18 (15.4)
Non diabetic	99 (85.6)
Anemic	116 (99.1)
Non anemic	1 (0.9)
Hypertension	114 (97.4)
Non HTA	3 (2.6)
Hepatitis B/C	
Positive	16 (13.7)
Negative	101 (86.3)
Number of comorbidities	
1–2	76 (65.0)
≥3	41 (35.0)
Hemodialysis access	
Arteriovenous Fistula/ Graft (AVF/AVG)	71 (60.7)
Temporary Femoral Vein Catheter (FVC)	11 (9.4)
Temporary Jugular Vein Catheter (JVC)	17 (14.5)
Permanent dialysis catheter (PermaCath)	18 (15.4)

Regarding health scores the results showed the following: The Physical Component Summary (PCS) had a mean of (34.17 ± 12.99), with the lowest score in physical role limitations (28.80 ± 22.54) and the highest in physical function (37.01 ± 22.14). The Mental Component Summary (MCS) had a mean of 47.52 ± 13.95, with emotional role limitations scoring the lowest (36.6 ± 23.41) and emotional well-being the highest (52.73 ± 18.65). The Kidney Disease Component Summary (KDCS) had a mean of 49.84 ± 9.38, with the lowest score in the burden of kidney disease (15.21 ± 13.79) and the highest in dialysis staff encouragement (82.98 ± 18.04), followed by social support (75.32 ± 33.40), Table 2.

**Table 2:** KDOQL-SF scores for principal and sub domains.

Principal domain	Sub-domain	Mean ± SD
PCS mean score 34.17 ± 12.99	Physical function	37.01 ± 22.14
	Physical role	28.80 ± 22.54
	Pain	35.26 ± 23.19
	General health	35.60 ± 12.76
MCS mean score 47.52 ± 13.95	Emotional well-being	52.73 ± 18.65
	Role emotional	36.6 ± 23.41
	Social function	52.08 ± 17.94
	Energy/fatigue	48.67 ± 16.84
KDCS mean score 49.84 ± 9.38	Symptom/problem list	48.83 ± 17.44
	Effect of kidney disease	39.19 ± 17.94
	Burden of kidney disease	15.21 ± 23.79
	Work status	16.15 ± 12.84
	Cognitive function	58.83 ± 23.35
	Quality of social interaction	60.29 ± 20.85
	Sexual function	40.94 ± 30.50
	Sleep	42.28 ± 16.05
	Social support	75.32 ± 33.40
	Dialysis staff encouragement	82.98 ± 18.04
	Patient satisfaction	68.17 ± 29.49
Overall QoL mean score	Mean (PCS,MCS,KDCS)	45.95 ± 9.94

When considering different demographic and clinic factors, *female patients* had significantly lower scores in all domains, Physical Component Summary (PCS), Mental Component Summary (MCS) Kidney Disease Component Summary (KDCS) and Overall. Patients *over 60 years* old also scored lower in PCS, MCS, KDCS and Overall, compared to younger patients. *Unemployed patients* had higher MCS scores, while unmarried had higher MCS and Overall scores. University-educated patients had higher PCS scores, while those with diabetes had lower KDCS scores.

*Patients with three or more comorbidities* reported significantly lower quality of life scores in PCS, MCS and Overall compared to those with fewer comorbidities. Adding, Patients with a dialysis duration of more than 5 years had lower scores in the PCS (Table 3).

**Table 3:** Identification of associated factors affecting overall and the three domains of quality of life among hemodialysis patients.

Variables	Total number, *n* (%)	PCS		MCS		KDCS		Overall	
		Score	*p* value	Score	*p* value	Score	*p* value	Score	*p* value
		Median (IRQ)		Mean ± SD		Mean ± SD		Mean ± SD	
Gender									
Female	42 (35.9%)	31.2 (10.3)	0.047′(*)	41.7 ± 13.2	<0.0001^**a**^(***)	43.9 ± 8.1	<0.0001^**a**^(***)	39.6 ± 8.7	<0.0001^**a**^(***)
Male	75 (64.1%)	33.8 (17.5)		49.6 ± 11.9		52.8 ± 7.8		49.5 ± 8.8	
Age									
≤ 60 years	71 (60.7%)	33.8 (14.3)	0.011′(*)	49.2 ± 11.2	0.012^**a**^(*)	51.8 ± 8.2	<0.0001^**a**^(***)	48.4 ± 8.9	<0.0001^**a**^(***)
>60 years	46 (39.3%)	30.0 (13.1)		43.1 ± 14.6		46.3 ± 9.4		42.2 ± 10.4	
Occupation									
Unemployed	98 (83.8%)	31.3 (15.3)	0.242′	49.1 ± 13.4	0.002^**a**^(**)	49.5 ± 8.9	0.732^a^	45.9 ± 9.1	0.902^a^
Employed	19 (16.2%)	35.0 (18.1)		38.5 ± 12.2		50.3 ± 9.6		46.2 ± 13.6	
Residence									
Urban	73 (62.4%)	31.3 (14.2)	0.490′	48.3 ± 15.4	0.359^a^	50.7 ± 9.28	0.283^a^	46.2 ± 10.8	0.757^a^
Rural	44 (37.6%)	32.2 (17.1)		45.8 ± 10.5		48.8 ± 9.4		45.6 ± 8.4	
Education status									
Elementary	45 (38.5%)	28.6 (8.1)	<0.0001″(***)	43.6 ± 11.9	0.163^b^	48.6 ± 10.4	0.715^b^	44.6 ± 8.8	0.490^b^
High school	49 (41.9%)	37.5 (19.7)		50.0 ± 13.6		50.0 ± 9.2		46.2 ± 10.6	
Professional	13 (11.1%)	32.5 (9.4)		44.2 ± 8.6		51.1 ± 9.4		49.5 ± 11.6	
University	10 (8.5%)	43.8 (25.7)		51.0 ± 7.6		48 ± 6.3		48.0 ± 6.3	
Civil status									
Not married	28 (23.9%)	31.3 (9.8)	0.430′	52.3 ± 12.4	0.031^**a**^(*)	52.0 ± 9.2	0.115^a^	49.4 ± 9.5	0.033^**a**^(*)
Married	89 (76.1%)	33.2 (16.5)		45.9 ± 13.9		48.9 ± 8.9		44.8 ± 9.9	
Economic status									
Low	36 (30.8%)	31.3 (13.1)	0.901″	47.8 ± 0.00	0.730^b^	53.8 ± 8.1	0.152^b^	45.3 ± 9.5	0.888^b^
Middle	77 (65.7%)	32.5 (17.2)		47.8 ± 13.3		50.3 ± 9.5		46.3 ± 9.8	
High	4 (3.5%)	31.5(21.7)		46.0 ± 12.3		49.8 ± 0.0		45.3 ± 18.5	
Duration of dialysis									
≤5 years	67 (57.3%)	33.8 (16.3)	0.050′	48.5 ± 12.8	0.241^a^	49.6 ± 9.7	0.936^a^	47.1 ± 9.6	0.252^a^
>5 years	50 (42.7%)	31.3 (11.4)		45.6 ± 14.9		49.4 ± 9.1		44.4 ± 10.2	
Comorbidity									
Diabetic	18 (15.4%)	31.3 (15.6)	0.838′	47.3 ± 14.4	0.919^a^	48.5 ± 8.8	0.004^**a**^(**)	45.2 ± 6.4	0.642^a^
Non diabetic	99 (84.6%)	34.3 (16.6)		47.7 ± 9.9		55.4 ± 10.9		46.1 ± 10.1	
Anemic	116 (99.1%)	31.3 (16.3)	0.544′	44.6 ± 12.5	0.796^a^	51.2 ± 9.3	0.830^a^	45.9 ± 9.98	0.987^a^
Non anemic	1 (0.9%)	41.3 (0.00)		44.7 ± 0.00		52.4 ± 0.00		46.1 ± 0.00	
Hepatitis B/C (Positive)	16 (13.7%)	31.3 (16.3)	0.806′	44.6 ± 12.5	0.441^a^	51.0 ± 8.6	0.836^a^	45.4 ± 11.77	0.545^a^
Negative	101 (86.3%)	32.5 (18.9)		47.0 ± 12.3		51.6 ± 9.3		45.8 ± 9.67	
Number of comorbidities									
1–2	76 (65.0%)	32.3 (15.0)	0.024′(*)	49.3 ± 13.4	0.035^**a**^(*)	49.6 ± 9.2	0.986^a^	47.7 ± 10.0	0.006^**a**^(**)
≥3	41 (35.0%)	30.1 (15.6)		43.7 ± 13.9		49.0 ± 9.5		42.5 ± 9.0	

(^a^)t-test, (^b^)F-test, (′)Mann Whitney-U test, (″)Kruskal Wallis-test. *, ** and *** denote significance at the 5%, 1%, and 0.1%, respectively.

Referring to binary logistic regression the factors linked to poor physical quality of life (PCS) included being female, having a lower level of formal education (elementary/professional/high school), and having been on dialysis for more than 5 years.

Regarding mental health (MCS), married and employed patients had significantly worse quality of life. Diabetic patients were more likely to report poor kidney-related quality of life (KDCS), as were older and female patients (Table 4).

**Table 4:** Binary logistic regression analysis of associated factors affecting overall and components of quality of life among hemodialysis patients.

Regression PCS	B	Std. error	Wald	df	Sig.	Exp (B) OR	95% Confidence Interval for Exp (B)
							Lower—Upper Bound
Gender							
Female	2.15	1.05	4.14	1	0.042	8.59	1.08–68.25
Male	REF						
Age							
>60 years	0.17	0.59	0.09	1	0.769	1.19	0.37–3.81
≤ 60 years	REF						
Education status							
Elementary/professional/high	1.86	0.72	6.61	1	0.010	6.46	1.55–26.82
University	REF						
Civil status							
Not married	0.71	0.79	0.78	1	0.376	2.03	0.43–9.65
Married	REF						
Occupation							
Unemployed	0.39	0.70	0.31	1	0.577	1.48	0.37–5.91
Employed	REF						
Duration of dialysis							
>5 years	1.65	0.79	4.41	1	0.036	5.24	1.12–24.57
≤5 years	REF						
Comorbidity							
Diabetic	0.09	0.81	0.02	1	0.903	1.10	0.23–5.41
Non diabetic	REF						
Number of comorbidities							
≥3	1.29	0.79	2.69	1	0.101	3.66	0.77–17.21
1–2	REF						

Regression MCS	B	Std. Error	Wald	df	Sig.	Exp (B) OR	95% Confidence Interval for Exp (B)
							Lower—Upper
Gender							
Female	0.288	0.40	0.50	1	0.476	1.33	0.60–2.94
Male	REF						
Age							
>60 years	0.677	0.40	2.78	1	0.095	1.97	0.88–4.35
≤ 60 years	REF						
Education status							
Elementary/professional/high	0.55	0.66	0.70	1	0.402	1.74	0.47–6.40
University	REF						
Civil status							
Married	1.07	0.45	5.75	1	0.017	2.91	1.22–6.95
Not married	REF						
Occupation							
Employed	2.64	1.05	6.37	1	0.012	14.07	1.81–109.62
Unemployed	REF						
Duration of dialysis							
>5 years	0.42	0.39	1.16	1	0.281	1.53	0.71–3.29
≤5 years	REF						
Comorbidity							
Diabetic	0.61	0.52	1.37	1	0.238	1.83	0.66–5.21
Non diabetic	REF						
Number of comorbidities							
≥3	0.39	0.41	0.93	1	0.326	1.48	0.67–3.31
1–2	REF						

Regression KDCS	B	Std. Error	Wald	df	Sig.	Exp (B) OR	95% Confidence Interval for Exp (B)
							Lower—Upper
Gender							
Female	1.68	0.43	15.01	1	0.001	5.37	2.29–12.57
Male	REF						
Age							
> 60 years	1.42	0.41	12.07	1	0.001	4.14	1.86–9.22
≤ 60 years	REF						
Education status							
University	0.87	0.72	1.46	1	0.227	2.38	0.59–9.69
Elementary/professional/high	REF						
Civil status							
Married	0.84	0.45	3.48	1	0.062	2.308	0.96–5.56
Not married	REF						
Occupation							
Unemployed	0.19	0.50	0.14	1	0.709	1.206	0.45–3.23
Employed	REF						
Duration of dialysis							
>5 years	0.33	0.38	0.78	1	0.378	1.39	0.67–2.91
≤5 years	REF						
Comorbidity							
Diabetic	1.18	0.56	4.37	1	0.040	3.25	1.08–9.81
Non diabetic	REF						
Number of comorbidities							
≥3	0.18	0.37	0.24	1	0.628	1.19	0.58–3.38
1–2	REF						

Regression overall	B	Std. Error	Wald	df	Sig.	Exp (B) OR	95% Confidence Interval for Exp (B)
							Lower—Upper
Gender							
Female	1.61	0.49	10.37	1	0.001	4.97	1.88–13.21
Male	REF						
Age							
>60 years	0.97	0.43	5.06	1	0.025	2.63	1.13–6.13
≤ 60 years	REF						
Education status							
Elementary/professional/high	0.21	0.72	0.09	1	0.771	1.23	0.30–5.05
University	REF						
Civil status							
Married	0.69	0.44	2.41	1	0.121	1.99	0.83–4.75
Not Married	REF						
Occupation							
Unemployed	0.67	0.51	1.71	1	0.190	1.94	0.71–5.27
Employed	REF						
Duration of dialysis							
>5 years	0.65	0.41	2.57	1	0.109	1.92	0.86–4.27
≤5 years	REF						
Comorbidity							
Diabetic	0.69	0.60	1.31	1	0.251	2.00	0.61–6.54
Non diabetic	REF						
Number of comorbidities							
≥3	1.31	0.47	7.66	1	0.006	3.72	1.47–9.46
1–2	REF						

## Discussion

### Assessment of quality of life in study participants

ESKD is a life-threatening condition, and its treatment remains challenging (9, 11, 23). The findings from our study, which involved 117 participants, along with previous research, emphasize that the main goal of treatment is not to cure the disease but to enhance and preserve the patients’ quality of life (7, 24).

The study found that, in terms of QOL scores, physical role limitations had the lowest score (28.80 ± 22.54), while physical function had the highest (37.01 ± 22.14), resulting in a mean of 34.17 ± 12.99 in the Physical Component Summary (PCS), Table 2. A similar pattern was observed in other studies (11, 25). The limitations of physical activity and the low score on the burden of kidney disease subscale highlighted the negative impact on patients’ work and daily life, with many feeling like a burden to their families. Quality of life in ESKD has emerged as a crucial metric for assessing both the benefits and challenges of dialysis from the patient’s perspective, encompassing how they manage their overall health, as highlighted by other study (7). Furthermore, the findings of our study are consistent with those of other studies, which attribute this to factors such as dependence on medical support, weekly dialysis sessions, sleep disorders, immobility, and changes in body image (11, 26–28). In our study, as presented in Table 2, the Mental Component Summary (MCS) had an average score of 47.52 ± 13.95. Among the subdomains, emotional role limitations had the lowest score (36.6 ± 23.41), while emotional well-being scored the highest (52.73 ± 18.65). These results align with findings from other studies, which indicate that the progression of the illness adversely impacts both mental and physical health, often resulting in financial challenges and difficulties in interpersonal relationships (29).

The results for the Kidney Disease Component Summary (KDCS) had a mean score of 49.84 ± 9.38, with the burden of kidney disease scoring the lowest (15.21 ± 13.79) and dialysis staff encouragement the highest (82.98 ± 18.04), followed by social support (75.32 ± 33.40), as shown in Table 2. The highest scores for social support and dialysis staff encouragement in our study may be due to the close-knit social structure of Albanian society. Additionally, the overall QOL score observed was low. In this regard, an inconsistency was found with the literature, which may be attributed to differences in study methodologies, local cultures, social/economic welfare, and other confounders, as reported by similar research (30, 31). For example, our QOL score was higher than those reported by Shumbusho et al. (32) and Gebrie et al. (8), but lower than the scores reported by Joshi et al. (1) and Sharma et al. (33).

### Factors contributing to the quality of life related to the health of study participants

In this study, we also evaluated the influence of socio-demographic and clinical factors, such as comorbidities, on the quality of life among patients undergoing hemodialysis therapy. We hypothesized that there is a relationship between these variables. When considering demographic factors, female patients scored significantly lower on all domains, the Physical Component Summary (PCS) (31.2 (10.3), *p* = 0.047), Mental Component Summary (MCS) (41.7 ± 13.2, *p* < 0.0001), Kidney Disease Component Summary KDCS (43.9 ± 8.1, *p* < 0.0001) and Overall (39.6 ± 8.7, *p* < 0.0001). Patients older than 60 years also had lower PCS (30.0 (13.1), *p* = 0.011), MCS (43.1 ± 14.6, *p* = 0.012), KDCS (46.3 ± 9.4, *p* < 0.0001) and Overall (42.2 ± 10.4, *p* < 0.0001) scores compared to those aged 60 years or younger (Table 3). The results are similar to those of other studies that found female patients undergoing hemodialysis experienced a significant deterioration in all domains of health-related quality of life prior to starting dialysis except for older patients who had better results in the mental component (34, 35). In addition, in terms of age, as found by other research studies, older patients had significantly lower PCS, MCS and KDCS scores due to geriatric syndromes, such as negative experiences related to physical health, cognitive impairment, or lower life expectations (11, 14, 33). Regarding gender, our findings are similar to those of other studies, which have indicated that males have better quality of life (QOL) due to better social relationships, sexual activity, and support compared to females (36). Similarly, differences in lifestyle, such as greater physical activity in males, have been reported (7). However, the literature also includes studies that have found results contrasting with those of our study. For example, in different studies, gender has not been reported as a significant factor impacting quality of life (QOL), which can be explained by social differences across countries rather than disease-specific factors (1, 11, 37, 38).

As revealed by our study, unemployed patients had higher MCS scores (49.1 ± 13.4, *p* = 0.002), and unmarried patients scored higher on the MCS (52.3 ± 12.4, *p* = 0.031) than married ones and on Overall (49.4 ± 9.5, *p* = 0.033). University-educated patients had higher PCS scores (43.8 (25.7), *p* < 0.0001) compared to those with a lower level of formal education (Table 3).

In summary, based on these demographic factors, our study results suggest that education level is a predictor of quality of life (QOL). Participants with elementary/professional/high school education had significantly lower QOL scores in the PCS and MCS domains. In addition, similar studies support this finding, showing that more educated patients with end-stage kidney disease (ESKD) tend to have better QOL, as higher education improves understanding of the disease and adherence to dialysis sessions (8, 9). Even though a study in Greece reported that education had no impact on physical and mental QOL scores (39).

According to Table 3, *occupation* and *civil status* were significantly linked to the mental subscale of quality of life (MCS). Additionally, civil status was found to be associated with overall quality of life (QOL), yielding findings that are contrary to those in the literature (1, 40). As studies suggest, the main reasons for these findings are that employed and married individuals may have more burdens in the workplace, as well as additional obligations and responsibilities within the family, which increase stress and affect participants’ quality of life (33). On the other hand, researchers argue that a family environment is a source of support and seems to have a positive effect on the quality of life (QOL) among HD patients (40). Moreover, employment status provides financial stability, which is expected to meet the needs of HD patients, increase self-esteem, and reduce worries about the future, all of which contribute to better QOL (1).

As shown in Table 3, patients suffering from diabetes mellitus (48.5 ± 8.8; *p* = 0.004) had significantly lower KDCS scores, suggesting this condition worsens the impact of kidney disease. Previous studies have reported similar findings (11, 25, 27, 31). Adding 97% of patients undergoing hemodialysis also have hypertension as a comorbidity. Individuals with three or more comorbidities demonstrated lower scores in PCS (30.1 (15.6), *p* = 0.024), MCS (43.7 ± 13.9, *p* = 0.035), and Overall score (42.5 ± 9.0, *p* = 0.006) (Table 1). In this regard, the results align with the literature, as the presence of co-morbid diseases and an increase in their total number have been recognized as variables negatively associated with HRQOL (41, 42).

In Binary Logistic Regression in our study, factors associated with low PCS scores included being female (OR = 8.59, *p* = 0.042, CI = 1.08–68.25, Ref: male), having elementary/professional/high education (OR = 6.46, *p* = 0.010, CI = 1.55–26.82, Ref: university), and dialysis duration >5 years (OR = 5.24, *p* = 0.036, CI = 1.12–24.57, Ref: ≤5 years). Female patients and those with a lower level of formal education were more likely to have poor physical quality of life. For low MCS scores, married (OR = 2.91, *p* = 0.017, CI = 1.22–6.95, Ref: unmarried) and employed (OR = 14.07, *p* = 0.012, CI = 1.81–109.62, Ref: unemployed) patients had higher odds of poor mental quality of life. Diabetic patients (OR = 3.25, *p* = 0.040, CI = 1.08–9.81, Ref: non-diabetic), female patients (OR = 5.37, *p* = 0.001, CI = 2.29–12.57), older ≥ 60 years (OR = 4.14, *p* = 0.001, CI = 1.86–9.22, were more likely to have poor kidney disease-related quality of life. Additionally, older patients ≥60 years (OR = 2.63, *p* = 0.025, CI = 1.13–6.13), female patients (OR = 4.97, *p* = 0.001, CI = 1.88–13.21), and with ≥3 comorbidities (OR = 3.73, *p* = 0.006, CI = 1.47–9.46,) had a higher likelihood of poor overall quality of life (Table 4). Our study results, particularly regarding the number of comorbidities and quality of life scores, align with another study that found a higher number of comorbidities worsens the burden of kidney disease, leading to a decrease in patients’ quality of life (41).

While, our study found significant relationship between the Kidney Disease Component Summary and comorbidity and no significant relationship between the mental and physical components and comorbidity. In contrast, other studies have shown that hepatitis C and anemia negatively impact both the physical and mental health of HD patients. This can be attributed to the hypoxic condition caused by anemia and the infectious nature or complications of hepatitis C, which can affect not only physical function but also cognitive performance, mood, and psychological well-being (43).

The *duration of dialysis* was found to have a statistically significant impact only on the physical component of quality of life with lower PCS scores (31.3 (11.4), *p* = 0.050), and no observed effect on other components such as MCS, KDCS, or the Overall score. This contrasts with another study (1), which suggested that longer hemodialysis duration was linked to a decline in quality of life. This discrepancy may be explained by the fact that as patients undergo hemodialysis for a longer period, they may encounter challenges such as a repetitive routine, persistent fatigue, frustration, and difficulties managing treatment symptoms, all of which can negatively affect their quality of life, as noted by previous study (8).

Additionally, no significant statistical difference was found in terms of *access sites* and their impact on the three QOL domains in our study. However, a study examining the link between hemodialysis access type and patient satisfaction found that patients with arteriovenous (AV) fistulas had the highest satisfaction levels, which were tied to better HRQOL outcomes (44).

### Limitations and strengths of the study

This study has several key strengths. First, it utilized the KDQOL-36, a widely recognized and standardized tool for assessing health-related quality of life (HRQOL), which has been validated among Albanian CKD patients. This validation allows for meaningful comparisons with other studies conducted in different regions. Additionally, this research is the first to investigate HRQOL among CKD patients in Southern Albania, offering valuable insights into the quality of life for individuals undergoing treatment. The findings could encourage healthcare professionals in nephrology and social care units to enhance dialysis services in the future. Moreover, the results may help doctors, medical staff, and family members better understand the physical and psychological challenges faced by patients on maintenance hemodialysis (MHD), enabling them to provide more effective support.

Despite these strengths, the study has several limitations. Its cross-sectional design prevents the establishment of cause-and-effect relationships. Furthermore, the face-to-face interview method used for data collection may have introduced biases, including interviewer influence and social desirability bias. A notable limitation is the absence of an analysis of how biochemical factors impact the quality of life of CKD patients. Additionally, the study did not explore the role of medications in influencing HRQOL. Incorporating clinical variables such as albumin, calcium, and creatinine levels would have offered a more comprehensive understanding of the factors affecting dialysis outcomes and HRQOL in hemodialysis patients.

## Conclusion

The study concludes that hemodialysis significantly impacts the health-related quality of life (HRQOL) of patients with chronic renal failure in Southern Albania. It was found that HRQOL is notably impaired, with the lowest scores observed among women, older individuals, those with a lower level of formal education, unemployed patients, married individuals, those undergoing dialysis for more than 5 years, and those with co-morbid diseases. As the total number of co-morbidities increases, HRQOL further decreases. This evidence underscores the vulnerability of these patient groups and highlights the need for specific interventions to improve their quality of life. It also calls for improved resource allocation in dialysis units, including better staffing, counseling services, and more patient-friendly facilities to enhance care delivery.

Furthermore, the study revealed that the physical component summary scores were much lower than the mental and KDCS components. This finding emphasizes the physical toll that both the disease and the treatment process have on patients, suggesting that physical health aspects should be prioritized when addressing the challenges faced by individuals undergoing hemodialysis.

The results also point to the necessity for targeted interventions to improve HRQOL in this population. This is especially important for groups such as women, older patients, and those with longer durations of dialysis and co-morbid conditions, who are most at risk of poor HRQOL. The evidence suggests that addressing the physical, psychological, and social needs of these patients through an integrated care approach is essential.

Finally, the study concludes by recommending continued research to better understand the long-term effects on HRQOL among hemodialysis patients in Albania. This includes evaluating the effectiveness of various interventions to improve the well-being of patients undergoing hemodialysis.

## Data Availability

The original contributions presented in the study are included in the article/supplementary material, further inquiries can be directed to the corresponding author.
